# Synthesis of ZnO Nanomaterials Using Low-Cost Compressed Air as Microwave Plasma Gas at Atmospheric Pressure

**DOI:** 10.3390/nano9070942

**Published:** 2019-06-28

**Authors:** Byeong-Joo Lee, Sung-Il Jo, Goo-Hwan Jeong

**Affiliations:** Department of Advanced Materials Science and Engineering, Kangwon National University, Chuncheon, Gangwon-do 24341, Korea

**Keywords:** zinc oxide, nanowire, microwave plasma, atmospheric pressure, compressed air

## Abstract

Zinc oxide (ZnO) nanomaterials were efficiently synthesized using a microwave plasma torch system at atmospheric pressure. The Zn powder was passed through a microwave plasma region, in which it melted and vaporized. Tetrapod-type ZnO nanomaterials with a diameter of 29.8 ± 8.0 nm were synthesized using a high-purity O_2_/N_2_ mixed gas. In particular, ZnO nanowires with a diameter of 109.5 ± 8.0 nm and a length of 5–6 μm were produced using an inexpensive compressed air as a microwave plasma gas. It was confirmed that the nanowires synthesized using the compressed air showed higher light absorption in the visible region than the tetrapod-type ZnO. In addition, the redshifts in the absorption peak and photoluminescence peak were observed from 370.6 to 375.2 nm and 380 to 390 nm, respectively. The obtained results can be explained by the change of energy levels due to the defects in the ZnO nanowires such as vacancies and interstitials of Zn and oxygen. Finally, we can conclude that cost-effective compressed air is appropriate not only for the synthesis of ZnO nanowire, but also the enlargement of optical absorption and emission range.

## 1. Introduction

Zinc oxide (ZnO) has attracted much attention because it exhibits interesting physical properties as a representative wide bandgap semiconductor nanomaterial [1,2]. For this reason, ZnO nanostructures are of particular interest in electronic and optical applications, such as optoelectronic devices, energy harvesting devices, sensors, and catalysts [3,4,5]. From the point of view of industrial applications, a quick and cost-effective synthesis of ZnO nanomaterials with precise morphology is still highly desirable. 

Several methods for nanostructured-ZnO synthesis have been reported in the literature. Solid-phase synthetic methods are relatively inadequate for producing high quality ZnO nanomaterials because of the incorporation of impurities and the non-uniform aggregation of nanoparticles in the grinding process [6,7]. Liquid-phase synthetic approaches, such as direct precipitation, sol-gel, emulsion, and hydrothermal methods, suffer from large particle agglomeration due to reactions in the liquid phase [8,9,10]. Moreover, such liquid-phase methods are complicated, time consuming, and even harmful to the environment. On the other hand, vapor-phase synthesis is preferred because it shows a high reaction rate and produces high-purity ZnO. However, it takes a lot of energy to maintain a high temperature and a continuous process [11,12]. To overcome these disadvantages, a method for synthesizing ZnO nanomaterials in a short time using a high-frequency—such as microwave or radio-frequency—power source is proposed, including an atmospheric microwave plasma torch system [13,14]. Various particles such as nanorods, tripods, tetrapods, and nanoclusters have been synthesized using this method. The particle morphology can be controlled by changing the gas composition and mixing ratio, but it was still difficult to control the whole process [15,16]. In addition to morphology, it is important to consider the electronic band structure of ZnO nanomaterials owing to its wide bandgap character. The electronic band structure of ZnO is significantly affected by various defects, mainly Zn interstitial (Zn_i_), Zn vacancy (V_Zn_), oxygen interstitial (O_i_), and oxygen vacancy (V_O_) [17,18,19,20,21,22,23]. Thus, the control of morphology, as well as defects, is a very critical factor for the application of ZnO in industrial fields. 

In this study, an atmospheric-pressure microwave plasma apparatus was constructed and used to synthesize ZnO nanomaterial in a continuous process. A micro-sized Zn powder used as the starting material was instantly vaporized as it passed through the microwave plasma plume at atmospheric pressure. ZnO with tetrapod- and rod-shaped nanomaterials were produced in the chemical reaction of the plasma by using high-purity air, O_2_, and O_2_/N_2_ mixture gases. Meanwhile, ZnO nanowires were selectively synthesized when compressed air was used. The influences of structural differences, such as shape and defects, on the energy band structure were investigated by optical analysis via ultraviolet (UV)-visible spectrophotometry and photoluminescence (PL) studies. 

## 2. Materials and Methods

### 2.1. Atmospheric Pressure Microwave Plasma System

**Figure 1:**
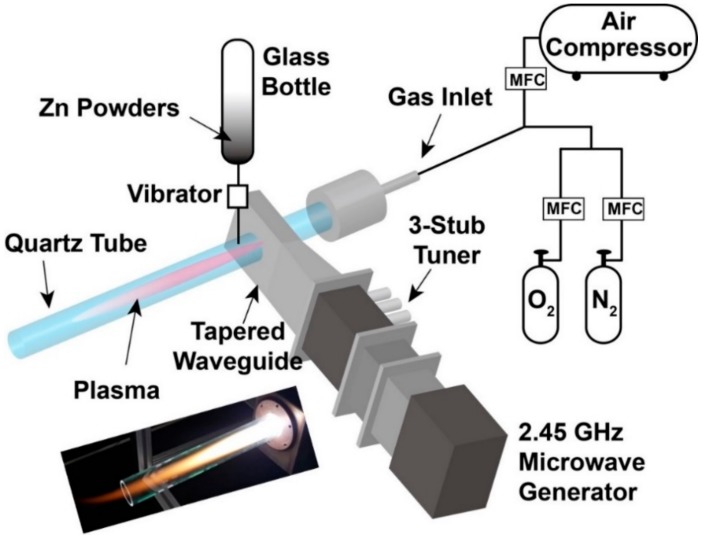
Schematic of microwave plasma system at atmospheric pressure.

The microwave plasma apparatus for ZnO synthesis consisted of a 2.45 GHz microwave generator (LG, 2M246, Busan, Korea), a tapered waveguide, and quartz tube with an inner diameter of 28 mm as shown in Figure 1. There is a 3-stub tuner between the microwave generator and the waveguide, which protects the generator by preventing reflection. As shown in Figure 1, the quartz tube is installed through the tapered waveguide and the atmospheric plasma is ignited in the quartz tube with high density and temperature. For a typical air plasma, the plasma density is about to 10^13^ cm^−3^ and the maximum plasma temperature is 6500 ± 350 K [24]. Thus, microwave plasma at atmospheric pressure provides a highly reactive chemical environment in which various reactions can take place.

### 2.2. Synthesis of ZnO Nanomaterials

At one end of the quartz tube, we devised a specific gas inlet for plasma gas swirl injection. All plasma gases including air, O_2_, and O_2_/N_2_ mixed gas (20/80 vol.%) are of high-purity (99.999%) except for compressed air. The flow rate of the gases was set to 10 lpm. A spherical Zn powder (Sigma-aldrich, 95% purity, 10 μm in diameter MO, USA) was the raw material and continuously inserted through a vibrator into the plasma plume in the quartz tube. The Zn powder reacts with the plasma, thereby synthesizing the ZnO nanomaterial. The synthesized ZnO was deposited on the inner wall of the quartz tube and collected using a scraper. The production yield of the ZnO was confirmed to be 4.0 g/h.

### 2.3. Characterization

The structural properties of the ZnO products were characterized by an X-ray diffraction (XRD, PANalytical, X’Pert Pro Royston, UK) using Cu K_α_ radiation (λ = 0.154 nm), scanning electron microscopy (SEM, Zeiss, Supra 55VP Oberkochen, Germany)), and transmission electron microscopy (TEM, Jeol, JEM-2100F Tokio, Japan). The optical absorption property was evaluated by using a UV-visible spectrophotometer (Biochrom, Libra S80 Selb, Germany) and PL measurement (Dongwoo Optron, MonoRa500i Gyeonggi-do, Korea) was performed at room temperature using a He-Cd laser (325 nm) with a power of 25 mW. 

## 3. Results and Discussion

**Figure 2:**
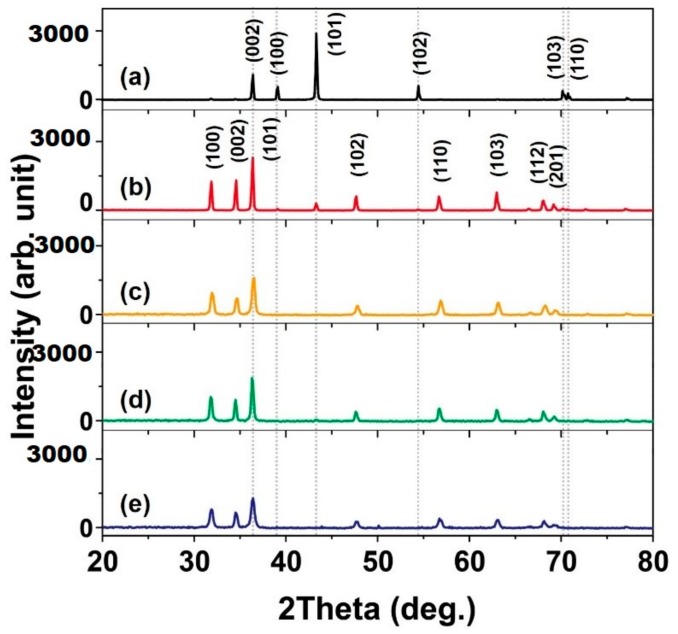
XRD spectra of (**a**) Zn powder and synthesized ZnO nanomaterials using (**b**) compressed air, (**c**) high-purity air, (**d**) O_2_, and (**e**) O_2_/N_2_ mixed gas.

We confirmed from the XRD results that the Zn powder reacted in the microwave plasma to synthesize ZnO. The Zn powder had a hexagonal crystal system of space group P63/mmc. The main peak of Zn powders is observed at 43.3° by the (101) plane, and peaks related to the (002), (100), and (102) planes are observed at 36.3, 39.0, and 54.3°, respectively (Figure 2a) [JCPDS file No. 04-0831 and 79-0205]. After the microwave plasma synthesis using compressed air, high-purity air, O_2_, and O_2_/N_2_ mixed gas, the Zn peaks indicated by the dotted lines in Figure 2 disappeared. Instead, strong peaks related to ZnO in the hexagonal type space group P63mc appeared at 31.8, 34.5, and 36.4°. This implies that ZnO nanomaterials were synthesized after the plasma reaction. The crystallite size of the synthesized ZnO nanomaterials was estimated using the Scherrer equation [25].

τ = *K*λ/βcosθ (1)

Here, *τ* represents the crystallite sizes, *β* is full width at half maximum (FWHM), *K* is a shape factor (usually 0.9) [26], and *λ* is the wavelength of the X-ray source. The crystallite sizes of the synthesized ZnO nanomaterials using compressed air, high-purity air, O_2_, and O_2_/N_2_ mixed gas were calculated to be 39.7, 23.0, 31.2, and 21.9 nm, respectively. The crystallite size of nanorods and tetrapods synthesized with compressed air and O_2_ was larger than that of high-purity air and O_2_/N_2_ mixed gas. 

**Figure 3:**
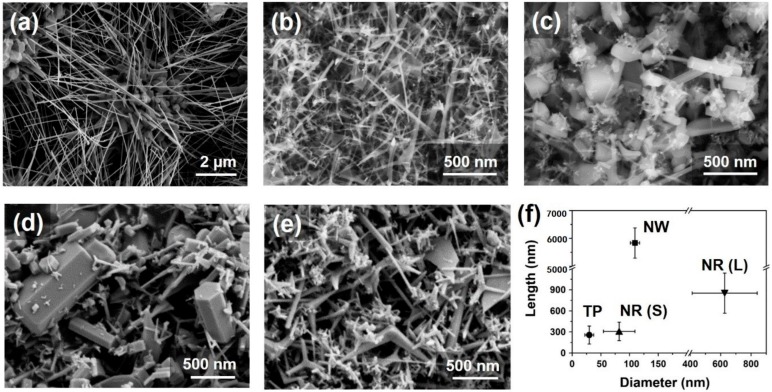
SEM images of synthesized ZnO nanomaterials using (**a**), (**b**) compressed air, (**c**) high-purity air, (**d**) O_2_, and (**e**) O_2_/N_2_ mixed gas. (**f**) Diagram shows length versus diameter of synthesized ZnO nanomaterials (NW; nanowire, TP; tetrapod, and NR (S), (L); small and large nanorod).

To examine the morphology and size distribution of the ZnO synthesized using compressed air, high-purity air, O_2_, and O_2_/N_2_ mixed gas, we performed SEM observation. When compressed air was used, two types of ZnO, nanowire and tetrapod, were synthesized as shown in Figure 3a,b. In the case of high-purity air (Figure 3c) and O_2_ (Figure 3d), most of the ZnO was synthesized as nanorods with a small amount being tetrapod. The diameter of the ZnO nanorods synthesized with high-purity air (82.0 ± 27.5 nm) is smaller than that of ZnO from high-purity O_2_ plasma (626.5 ± 213.7 nm). When O_2_/N_2_ mixed gas was used, most of the ZnO synthesized was tetrapodic, as shown in Figure 3e. In addition, we quantitatively summarized the morphology, ratio, and size of the synthesized ZnO nanomaterials for each plasma gas as given in Table 1. The ratio of the ZnO nanomaterial morphology was estimated by counting the 120 particles from SEM images at each condition. Although nanowires, tetrapods, and nanorods were synthesized under all conditions, the most synthesized particle ratios were written in Table 1. 

It has also been reported that ZnO nanomaterials of various morphologies are synthesized depending on the process temperature and additives in the liquid phase process [9,27]. It is therefore assumed that the morphology of ZnO would vary with the type of gas molecules in the plasma used. Specifically, compressed air generally contains a large amount of moisture and various trace gases in addition to oxygen and nitrogen. Hydrogen contained in these gases is also known to play a key role in increasing the plasma temperature [28]. For this reason, the morphology of the synthesized ZnO nanomaterial varies due to the difference in the plasma temperature, which depends on the type of gas used.

**Figure 4:**
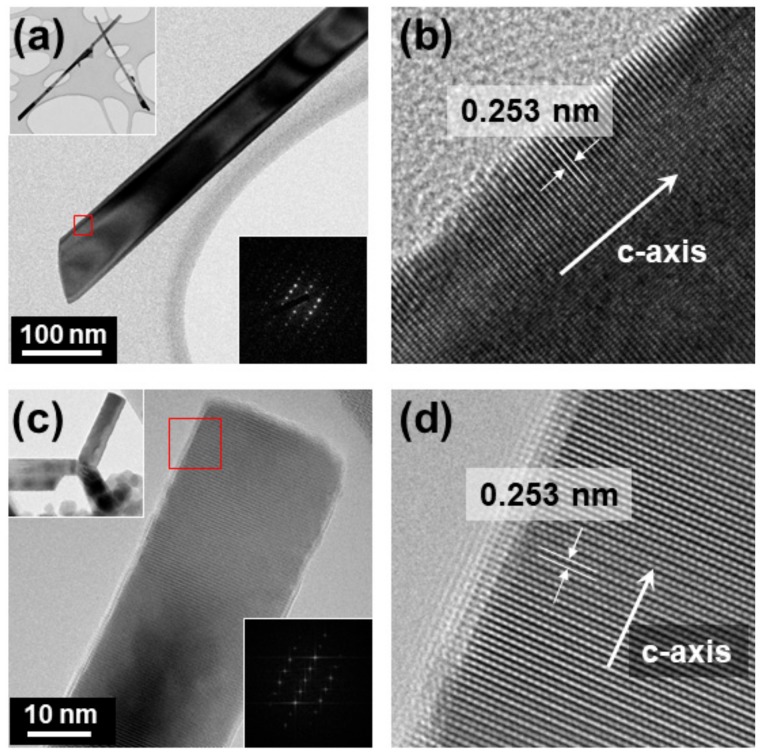
(**a**) TEM image of ZnO nanowire (inset: low-magnification image and SAD pattern). (**b**) High-magnification TEM image of ZnO nanowire at square area indicated in (**a**). (**c**) TEM image of ZnO tetrapod (inset: low-magnification image and FFT pattern). (**d**) High-magnification TEM image of ZnO tetrapod at square area indicated in (**c**).

In addition to the morphology analysis using SEM, TEM was employed to confirm the detailed crystal structure of synthesized ZnO. The representative TEM results of the ZnO nanowires and tetrapods synthesized using compressed air are shown in Figure 4. The analyzed nanowires had a diameter of 71.5 nm and a length of 2.5 μm. The selected area electron diffraction (SAED) pattern analysis confirmed that single crystal ZnO nanowires of a hexagonal crystal structure and space group of P63mc were synthesized (Figure 4a). The interlayer distance was found to be 0.253 nm which is approximately half of the 0.518 nm lattice constant of ZnO [JCPDS file No. 79-0205] in Figure 4b, and it was confirmed that the axial direction of the wire was the C-axis direction of the crystal structure (Figure 4b). In the case of tetrapod-type ZnO, the diameter was measured to be 25.8 nm and the length ranged from 106–140 nm. Fast Fourier transform pattern (FFT) analysis confirmed that the tetrapod-type ZnO was also monocrystalline (Figure 4c). The interlayer distance was also measured to be 0.253 nm, and it was confirmed that the axial direction of each pod was the C-axis direction of the crystal structure (Figure 4d).

**Figure 5:**
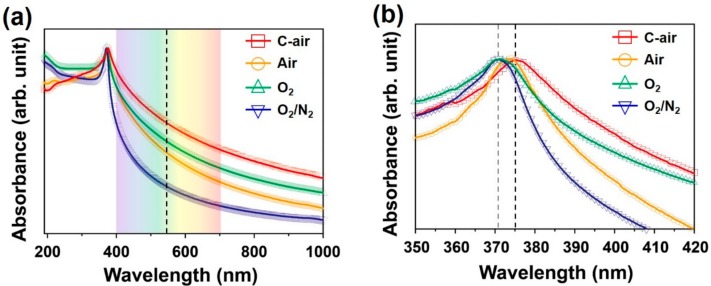
(**a**) Wide-range and (**b**) narrow-range UV-visible absorption spectra of ZnO nanomaterials synthesized using different gases.

The influence of the ZnO morphology on the energy band structure, which is important for any practical application, was determined by optical absorption spectroscopy and PL spectroscopy. The optical absorption spectra of the ZnO synthesized using compressed air, high-purity air, O_2_, and O_2_/N_2_ mixed gas are shown in Figure 5. All of the synthesized ZnO nanomaterials have a UV absorption near 370 nm, but a difference was observed at 400–700 nm in the visible region. ZnO nanowires synthesized using low-purity compressed air demonstrated higher absorption in the visible region than the tetrapod-type ZnO synthesized using high-purity O_2_/N_2_ mixed gas (Figure 5a). In addition, the position of the absorption peak was observed to shift from 370.6 to 375.2 nm when compressed air was used compared with when high-purity O_2_ and O_2_/N_2_ mixed gas was used (Figure 5b). This redshift in absorption peak can be attributed to a change in energy level by the formation of defects caused by the compressed air. The structural defect such as vacancies and interstitials would be formed during ZnO synthesis due to the various trace gases contained in the compressed air.

**Figure 6:**
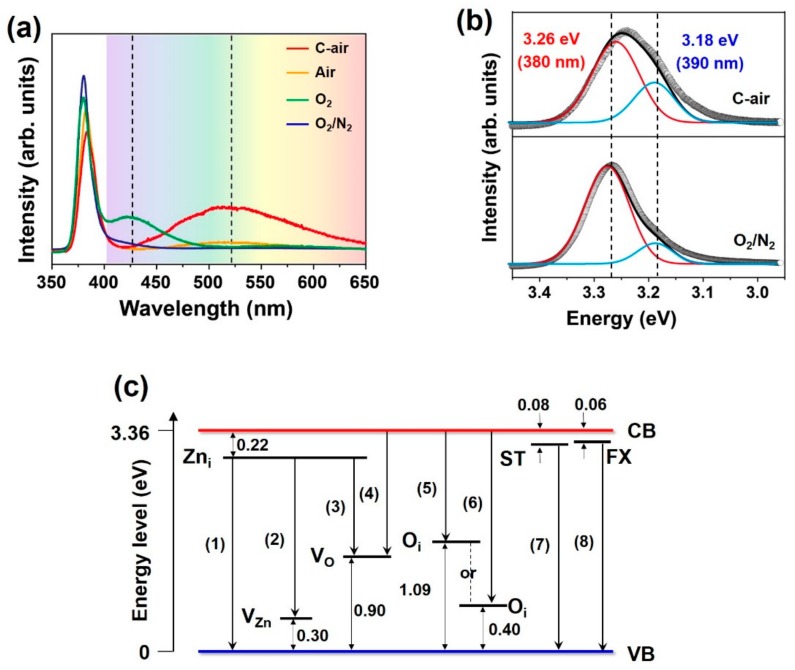
(**a**,**b**) Room-temperature PL spectra of synthesized ZnO nanomaterials using different gases. (**c**) Energy level diagram showing some of the principal defect levels in ZnO [18,19,20,21,22,23].

A PL analysis of the synthesized ZnO nanomaterials was performed to clarify the relationship between the optical properties and the energy level. The results are shown in Figure 6a,b. Figure 6c shows various energy levels and transitions in the energy level diagram of ZnO. The PL spectra in the visible region of ZnO synthesized using compressed air and high-purity O_2_ showed broad peaks near 500–550 nm and near 425 nm, respectively (Figure 6a). It is generally well known that transitions from Zn_i_ to V_O_ (2.24 eV, 554 nm), conduction band (CB) to V_O_ (2.46 eV, 504 nm) and CB to O_i_ (2.28 eV, 544 nm) (transitions (3), (4), and (5) in Figure 6c, respectively) as well as transitions from Zn_i_ to V_Zn_ (2.84 eV, 437 nm) and CB to O_i_ (2.90 eV, 419 nm) (transition (2) and (6) in Figure 6c, respectively) are related by energy levels Zn_i_, V_Zn_, O_i_, V_O_, and others, formed by defects such as vacancies and interstitials [18,19,20,21,22,23]. As mentioned, the low-purity compressed air is believed to have caused the formation of such defects. In the case of high-purity O_2_, it is considered that not only the defect formation, but also the size and morphology of synthesized large nanorods having a diameter of several hundred-nanometers, are believed to have caused the change of optical properties.

The peak in the UV region of ZnO synthesized using compressed air was red-shift compared with synthesized ZnO by using high-purity O_2_/N_2_ mixed gas. It is believed that this resulted from the formation of energy levels caused by structural defects in ZnO. Peak fitting confirmed that the red-shift was due to changes in the intensity of sub-peaks at 3.26 eV (380 nm) and 3.18 eV (390 nm) (Figure 6b). The sub-peak near 3.26 eV is due to transitions from the surface trap (ST) level to the valence band (VB) (3.28 eV, 378 nm) and from the free exciton (FX) level to the VB (3.30 eV, 376 nm) (transition (7) and (8) in Figure 6c, respectively) [29]. The sub-peak near 3.18 eV is due to a transition from Zn_i_ to the VB (3.14 eV, 395 nm) (transition (1) in Figure 6c) [30]. It was confirmed that the peak intensity at 390 nm was higher when compressed air was used than when high-purity O_2_/N_2_ mixed gas was used. This could be also attributed to the formation of defects primarily by the low-purity compressed air. It is worth emphasizing once again that ZnO nanowires synthesized using compressed air show enhanced PL intensity in the visible region and red-shift of peak in the UV region. 

## 4. Conclusions

We demonstrated the synthesis of ZnO nanomaterials by a microwave plasma system at atmospheric pressure using compressed air, high-purity air, O_2_, and O_2_/N_2_ mixed gas. ZnO nanowires were grown using low-cost compressed air with a diameter of 109.5 ± 8.0 nm and a length of 5–6 μm, while tetrapod-type ZnO with a diameter of 29.8 ± 8.0 nm was synthesized using high-purity O_2_/N_2_ mixed gas. Interestingly, the ZnO nanowires show enhanced light absorption at wavelengths of 380 nm and 500–550 nm as well as PL intensity in the visible region including red-shift of peak in the UV region. It may be concluded that the results were caused by the changes in electronic energy level due to the formation of defects in the ZnO nanowire. 

## Figures and Tables

**Table 1:** Morphology, ratio, and size of synthesized ZnO nanomaterials depending on the type of plasma gas.

Plasma Gas	Flow Rate [lpm]	Product	Ratio (%)	Size (nm)
Compressed air	10	Nanowire	69.2	Diameter = 109.5 ± 8.0
				Length = 5835.0 ± 543.2
		Tetrapod	25.0	Diameter = 29.8 ± 7.7
				Length = 256.5 ± 128.0
High-purity air	10	Small Nanorod	86.7	Diameter = 82.0 ± 27.5
				Length = 308.5 ± 131.8
High-purity O_2_	10	Large Nanorod	89.2	Diameter = 626.5 ± 213.7
				Length = 852.6 ± 286.2
High-purity O_2_/N_2_ mixed gas	O_2_ = 2	Tetrapod	92.5	Diameter = 29.8 ± 7.7
	N_2_ = 8			Length = 256.5 ± 128.0
